# Vitamin D Receptor Activation Mitigates the Impact of Uremia on Endothelial Function in the 5/6 Nephrectomized Rats

**DOI:** 10.1155/2010/625852

**Published:** 2010-02-10

**Authors:** J. Ruth Wu-Wong, William Noonan, Masaki Nakane, Kristin A. Brooks, Jason A. Segreti, James S. Polakowski, Bryan Cox

**Affiliations:** ^1^Department of Pharmacy Practice, University of Illinois at Chicago, Chicago, IL 60612-7230, USA; ^2^Abbott Laboratories, Renal Care, Abbott Park, IL 60048, USA; ^3^VidaGene, VDR Project, Chicago, IL 60612, USA

## Abstract

Endothelial dysfunction increases cardiovascular disease risk in chronic kidney disease (CKD). This study investigates whether VDR activation affects endothelial function in CKD. The 5/6 nephrectomized (NX) rats with experimental chronic renal insufficiency were treated with or without paricalcitol, a VDR activator. Thoracic aortic rings were precontracted with phenylephrine and then treated with acetylcholine or sodium nitroprusside. Uremia significantly affected aortic relaxation (−50.0 ± 7.4% in NX rats versus −96.2 ± 5.3% in SHAM at 30 *μ*M acetylcholine). The endothelial-dependent relaxation was improved to –58.2 ± 6.0%, –77.5 ± 7.3%, and –90.5 ± 4.0% in NX rats treated with paricalcitol at 0.021, 0.042, and 0.083 *μ*g/kg for two weeks, respectively, while paricalcitol at 0.042 *μ*g/kg did not affect blood pressure and heart rate. Parathyroid hormone (PTH) suppression alone did not improve endothelial function since cinacalcet suppressed PTH without affecting endothelial-dependent vasorelaxation. N-omega-nitro-L-arginine methyl ester completely abolished the effect of paricalcitol on improving endothelial function. These results demonstrate that VDR activation improves endothelial function in CKD.

## 1. Introduction

Chronic kidney disease (CKD) patients experience a high mortality rate from cardiovascular (CV) diseases [1, 2]. Endothelial dysfunction—characterized by a disruption in the delicate balance among vasodilation, oxidative stress, inflammation, thrombosis, and fibrinolysis maintained by the endothelium—has been linked to increased cardiovascular disease (CVD) risk [3–6]. Endothelial dysfunction is commonly observed in CKD, likely preceding other cardiovascular complications [7]. Not only do changes in renal endothelial function affect the progression of renal disease, but also systemic endothelial dysfunction significantly contributes to the severity of cardiovascular complications in CKD. CKD patients usually have hypertension [8] and/or diabetes [9], two of the conventional cardiovascular risk factors that are associated with endothelial dysfunction. In addition, likely other renal specific mechanisms may contribute to endothelial dysfunction in CKD. In early CKD one of the first serum parameters to be altered is a decrease in the serum 1,25-dihydroxyvitamin D_3_ levels [10], which raises the question of whether deficient VDR activation may be one of the renal specific risk factors for endothelial dysfunction in CKD. In addition, preclinical studies show that VDR may be involved in modulating smooth muscle cell proliferation/differentiation, the renin-angiotensin system, inflammation, thrombosis, and fibrinolysis; many of these factors are involved in endothelial function [11, 12]. 

Vitamin D_3_ is made in the skin, but requires activation into the active hormone, 1,25-dihydroxyvitamin D_3_ (1*α*,25(OH)_2_D_3_, calcitriol), for its proper functions. Calcitriol exerts its functions via binding to a nuclear receptor, the vitamin D receptor (VDR). Calcitriol and its analogs such as paricalcitol (19-nor-1*α*,25(OH)_2_D_2_) that activate vitamin D receptor (VDR) are commonly used to manage hyperparathyroidism secondary to CKD [13]. Numerous clinical observations show that VDR activation therapy was associated with a survival benefit in CKD patients with an effectiveness order of: paricalcitol > calcitriol > no VDR activation therapy, independent of the parathyroid hormone (PTH) and calcium levels; the survival benefit of VDR activation agents is likely associated with a decrease in cardiovascular-related mortality [14–18]. The mechanism(s) of action for the cardiovascular and survival benefit of VDR activation agents in CKD is still not well understood. 

VDR has been identified in more than 30 different tissues in the human body including the vasculature, and emerging evidence suggests that VDR may play a role in modulating cardiovascular functions [19]. Vitamin D and vitamin D analogs that activate VDR (VDR agonists or activators, VDRAs) have been shown to modulate inflammation, thrombosis, and vasolidation, which are some of the important risk factors associated with endothelial dysfunction [20]. However, it is not well studied how VDRAs affect endothelial function in CKD. The 5/6 nephrectomized (NX) uremic rat model is a useful model for studying renal insufficiency and has been used extensively for the evaluation of VDRAs on modulating serum calcium and PTH. Besides having elevated PTH, the 5/6 uremic rats develop endothelial dysfunction [21], left ventricular hypertrophy [22], and proteinuria [23]. In this study, we employed paricalcitol to investigate whether VDR activation mitigates the detrimental effect of uremia on endothelial function in the 5/6 NX rats. Our results demonstrate that uremia significantly compromises endothelial function while paricalcitol improves endothelial function in uremic rats in a dose-dependent manner independent of serum PTH levels or blood pressure.

## 2. Methods

### 2.1. Materials

Paricalcitol (19-nor-1*α*, 25-dihydroxyvitamin D_2_, 19-nor-1*α*, 25(OH)_2_D_2_), and cinacalcet (N-[1-(R)-(−)-(1-naphthyl)ethyl]-3-[3-(trifluoromethyl)phenyl]-1-aminopropane hydrochloride) were provided by Abbott Laboratories. Other reagents were of analytical grade.

### 2.2. Subtotally Nephrectomized Rats

The investigation conforms with the Guide for the Care and Use of Laboratory Animals published by the US National Institutes of Health (NIH Publication No. 85-23, revised 1996), and all experiments were approved by the Abbott Laboratories Internal Animal Care and Use Committee. Male, Sprague-Dawley rats were obtained from Charles River. The nephrectomy was performed on rats weighing ~200 g with a standard two-step surgical ablation procedure [24]. Rats were maintained on the Teklad 8640 diet containing 1.13% Calcium and 0.94% Phosphorus. Elevated creatinine levels confirmed uremia for all 5/6 nephrectomized rats.

### 2.3. Vascular Function Studies

Rats of established uremia were studied at Week 6 after surgery. In the paricalcitol studies, 5/6 nephrectomized rats received vehicle (5% EtOH/95% propylene glycol; 0.4 mL/kg; i.p.) or paricalcitol (doses as indicated) 3x/wk over a period of 12 days (6 doses total), and blood was drawn on Days 0 (24 hours before the first dose of treatment) and 13 (24 hours after the last dose of treatment), and assayed for creatinine, BUN, PTH, total calcium (Ca) and/or ionized Ca (iCa), and phosphorus (Pi). In the cinacalcet studies, uremic rats were treated with vehicle (Cavitron, 1.65 mL/kg; p.o) or cinacalcet (30 mg/kg; p.o.) daily for 13 days, and blood was drawn on days 0 and 13 (2 hours postdose) for testing. Untreated, age-matched SHAM rats served as controls. In some studies, untreated uremic rats were compared with vehicle-treated uremic rats to investigate the effect of vehicle. Rats were anesthetized with Nembutal (100 mg/kg). Thoracic aortas were excised, cut into sections and an aortic ring suspended in 10 mL tissue baths under 0.5 grams of resting tension in a modified Krebs solution containing (in mmol/L) 120 NaCl, 20 NaHCO_3_, 11 dextrose, 4.7 KCl, 2.5 CaCl_2_, 1.5 MgSO_4_, and 1.2 KH_2_PO_4_ equilibrated with 5% CO_2_–95% O_2_ (pH 7.4 at 37°C). Tension was recorded with Grass FT03 isometric transducers (Astro-Med, West Warwich, RI) connected to a Grass 7D polygraph and a Ponemah data acquisition system (Data-Sciences Intl, St. Paul, MN). Aortas were sensitized by addition of phenylephrine (PE, 3 *μ*M) with 10-minute washouts between intervals. Aortas were precontracted with PE (3 *μ*M), and the endothelium-dependent vasodilator acetylcholine (ACh) was added in half-log increments (1 nM–30 *μ*M) at 3–5 minute intervals, allowing time for the effect of ACh to plateau. After a 60-minute washout, aortas were precontracted with PE (3 *μ*M) and subsequently treated with endothelial-independent vasodilator sodium nitroprusside (SNP; 1 nM–1 *μ*M) at 3–5 minute intervals, allowing time for the effect to plateau. In a separate study, SHAM and 5/6 NX rats were treated with vehicle or paricalcitol at 0.16 *μ*g/kg and aortic rings were preincubated with 100 *μ*M L-NAME (N-omega-nitro-L-arginine methyl ester) for 1 hour prior to the addition of PE to induce contraction. Due to the limited availability of the tissue bath chambers, different treatments were independently given to different groups of uremic rats in order to properly prepare the aortic rings for relaxation studies.

### 2.4. Measurement of PTH and Serum Mineral Levels

Serum PTH was measured using a rat intact PTH ELISA kit obtained from ALPCO/Immutopics, Inc. (Windham, NH). Serum Ca, Pi, creatinine, and BUN concentrations were measured using an Abbott Aeroset. Blood iCa was determined using an i-STAT portable clinical analyzer with an ^EG^7+ cartridge.

### 2.5. Measurement of Blood Pressure

In a separate study, male Sprague-Dawley 5/6 nephrectomized rats at ~4 weeks after nephrectomy were surgically implanted with transmitters. Briefly, animals were anesthetized with 3% isoflurane, and a flexible catheter attached to a TA11PA-C40 radio transmitter (Data Sciences, St. Paul, MN ) was inserted in the abdominal aorta just below the renal arteries, the procedure as recommended by Data Sciences International [25]. The transmitter was secured to the abdominal muscle and remained in the abdominal cavity for the duration of the experiment. After surgery, rats were housed in individual cages positioned over an RPC-1 radiotelemetry receiver (Data Sciences, St. Paul, MN). Rats received food and water ad libitum. Hemodynamic data were collected and summarized daily. Rats were allowed to recover for 2 weeks postsurgery prior to onset of dosing. Rats at 6 weeks after nephrectomy were treated with vehicle (5% EtOH/95% propylene glycol; 0.4 mL/kg; i.p.) or paricalcitol at 0.042 *μ*g/kg, 3x/week for 14 days. SHAM rats treated with vehicle were used as control. One group of rats was given vehicle (i.p., 3x/week) plus enalapril (10 mg/kg, p.o., via drinking water, daily for 8 days, then switched to 30 mg/kg for another 6 days. The 30 mg/kg enalapril treatment did not further reduce blood pressure).

### 2.6. Data Analysis

For serum chemistry data, group mean ± SEM are presented. Differences between SHAM and uremic rats with different treatments were assessed using a one-way ANOVA followed by a Dunnett's post-hoc test. A paired t-test was used to assess differences between baseline Day 0 (before treatment) and Day 13 (after treatment) or as indicated. For vascular function, ACh and SNP-induced relaxations were calculated as the % of relaxation of the PE-induced precontraction. Differences in vascular function were determined using a two-way ANOVA, followed by a Bonferonni post-hoc test.

## 3. Results

### 3.1. Effect of Uremia and Paricalcitol Treatment on Endothelial Function and Blood Chemistry

**Figure 1:**
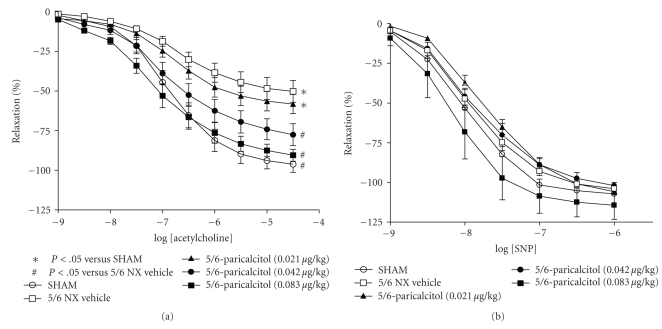
Endothelial dysfunction in 5/6 nephrectomized rats and the effect of paricalcitol. SHAM and 5/6 NX rats were treated with vehicle or paricalcitol at indicated doses as described in “Materials and Methods” (*n* = 9–11 per group). Aorta rings were precontracted with phenylephrine (PE, 3 *μ*M), and the endothelium-dependent vasodilator acetylcholine was added in half-log increments (1 nM–30 *μ*M) at 3–5 minute intervals. Afterwards, aorta rings were precontracted with PE (3 *μ*M) and subsequently treated with endothelial-independent vasodilator sodium nitroprusside (SNP, 1 nM–1 *μ*M) at 3–5 minute intervals. (a) Acetylcholine-evoked relaxation. (b) SNP-evoked relaxation.

We examined the effect of uremia and paricalcitol treatment on acetylcholine-induced endothelial-dependent relaxation of aortic rings from SHAM and 5/6 NX rats. As shown in Figure 1(a), the maximal aorta relaxation response to acetylcholine in SHAM rats was −96.2 ± 5.3% with an EC_50_ of 137 nM. Relaxation was significantly reduced in uremic 5/6 NX vehicle-treated rats (−50.3 ± 7.0%, *P* <.01, with an EC_50 _ of 211 nM). There was no difference in the relaxation response between vehicle-treated and untreated uremic rats (data not shown). The altered response to acetylcholine in the 5/6 NX rat indicates compromised endothelial function. As shown in Figure 1(b), the vascular relaxation produced by SNP (endothelial-independent vasodilator) was not significantly different between the SHAM and 5/6 NX rats, indicating that the aorta vascular smooth muscle relaxant response is intact and functional. 

Figure 1(a) also shows that 2-week treatment with paricalcitol produced dose-dependent improvement in acetylcholine-induced endothelial-dependent relaxation. Treatment with 0.083 *μ*g/kg of paricalcitol increased the maximal relaxation response to acetylcholine to −90.5 ± 3.8% (*P* <.05) with an EC_50_ at 75 nM. Treatment with 0.042 *μ*g/kg of paricalcitol shifted the EC_50_ to 121 nM, and increased the maximal relaxation response to acetylcholine to −77.5 ± 6.9% (*P* <.05). To a lesser degree, treatment with 0.021 *μ*g/kg of paricalcitol increased the maximal relaxation response to acetylcholine to −58.2 ± 6.0% with an EC_50_ at 105 nM. Figure 1(b) shows that paricalcitol had no significant effect on SNP-induced endothelial-independent relaxation. 

**Figure 2:**
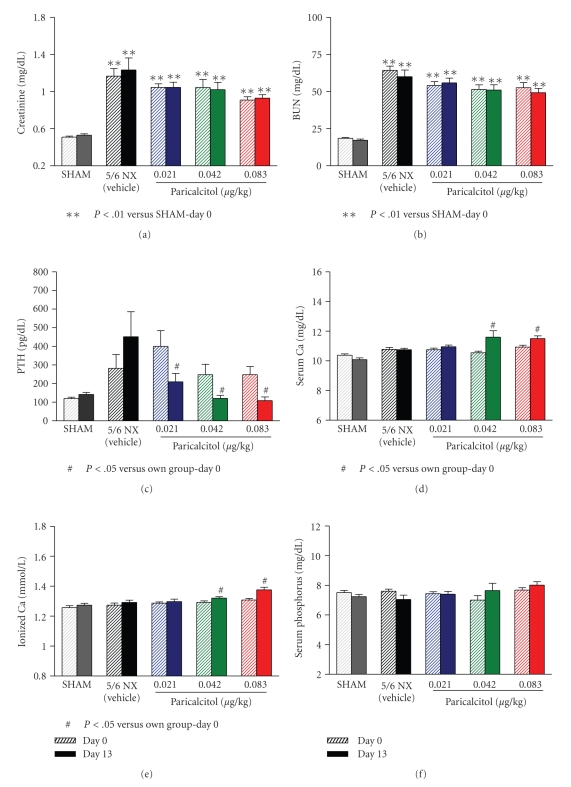
The blood chemistry in 5/6 NX uremic rats before and after paricalcitol treatment. The experimental conditions were as described in Figure 1. On Days 0 and 13 (24 hours after the last drug treatment), blood samples were collected for the measurement of (a) serum creatinine, (b) BUN, (c) serum PTH, (d) serum Ca, (e) ionized Ca, and (f) serum Pi. Mean ± standard error was calculated for each group (*n* = 9–11). One way ANOVA Dunnett test with 95% confidence intervals of difference was performed for statistical comparisons (Figures 2(a) and 2(b)).

As shown in Figures 2(a) and 2(b), the serum creatinine and BUN levels were significantly elevated in the 5/6 nephrectomized (NX) rats compared to SHAM rats, indicating a uniform disease state. Paricalcitol at the three tested doses had no significant effect on either creatinine or BUN (versus Day 0). Figure 2(c) shows that, as expected, paricalcitol effectively suppressed serum PTH in a dose-dependent manner (a reduction of 41.6 ± 7.2%, 41.6 ± 7.4% and 48.2 ± 8.3% at 0.021, 0.042 and 0.083 *μ*g/kg, resp.). Total serum calcium (Ca), phosphorus (Pi), and ionized Ca (iCa) levels were not significantly different in the 5/6 NX rats versus SHAM. Paricalcitol significantly increased serum Ca and iCa at 0.042 and 0.083 *μ*g/kg (Figures 2(d) and 2(e)). Paricalcitol at the three doses had no effect on the Pi levels (Figure 2(f)).

### 3.2. Paricalcitol Treatment Did Not Affect Blood Pressure and Heart Rate in 5/6 Nephrectomized Rats

**Figure 3:**
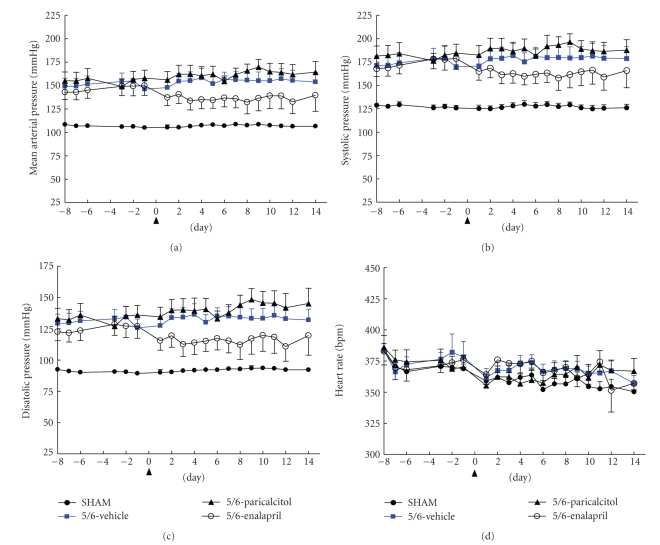
Effects of paricalcitol and enalapril on blood pressure and heart rate in 5/6 NX rats. SHAM or 5/6 NX rats were treated with vehicle or paricalcitol at 0.042 *μ*g/kg as described in “Materials and Methods” (*n* = 8–10 per group). SHAM rats treated with vehicle were used as control. One group of rats was given enalapril as another control. The arrow sign (Day 0) indicates the initiation of treatment. (a) Mean arterial pressure (MAP), (b) systolic pressure (SP), (c) diastolic pressure (DP), and (d) heart rate. MAP was 156.5 ± 7.8 mmHg in the 5/6 NX rat at Week 7 after surgery (i.e., Day 6 as shown in the graph) versus 107.1 ± 2.0 mmHg in SHAM; SP was 181.0 ± 10.0 versus 127.8 ± 3.8 mmHg; DP was 135.2 ± 6.6 versus 92.2 ± 1.5 mmHg.

To investigate whether paricalcitol improves endothelial function via lowering blood pressure, we examined blood pressure and heart rate in these animals. The angiotensin-converting enzyme (ACE) inhibitor enalapril was used as a control. Mean arterial pressure (MAP), systolic pressure (SP), and diastolic pressure (DP) were elevated in 5/6 NX rats (Figures 3(a)–3(c)). There was no significant difference in heart rate (Figure 3(d)). Enalapril exhibited a modest and stable effect on reducing mean arterial pressure, systolic pressure, and diastolic pressure, but had no effect on heart rate. As a comparison, paricalcitol at 0.042 *μ*g/kg had no significant effects on blood pressure or heart rate (Figure 3). Consistent with the study in Figure 2, paricalcitol had no significant effect on the creatinine, BUN, and serum Pi levels, while it effectively suppressed PTH and also significantly increased total serum Ca (data not shown). Enalapril did not show a significant effect on the creatinine, BUN, PTH, serum Ca, or Pi levels (data not shown). These results demonstrate that paricalcitol at 0.042 *μ*g/kg did not affect blood pressure, suggesting that the effect of paricalcitol at 0.042 *μ*g/kg on improving endothelial function is independent of blood pressure control.

### 3.3. PTH Suppression Alone Did Not Improve Endothelial Function

**Figure 4:**
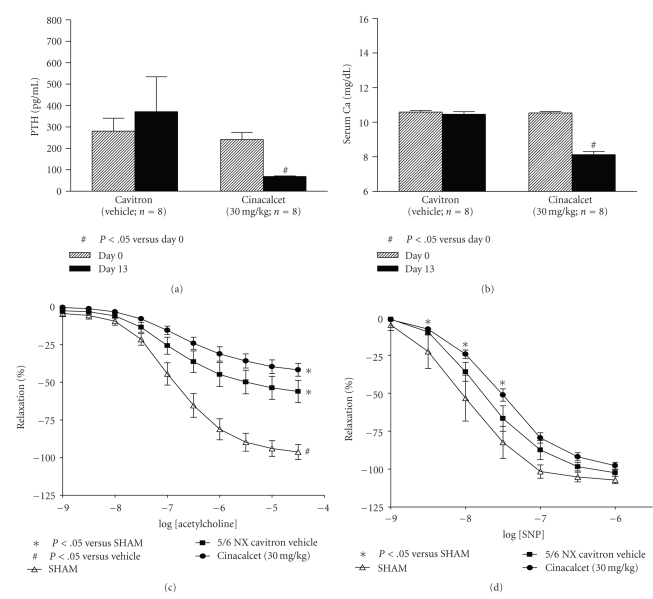
Cinacalcet suppressed PTH, but did not improve endothelial dysfunction in 5/6 nephrectomized rats. SHAM and 5/6 NX rats were treated with vehicle or cinacalcet as described in “Materials and Methods” (*n* = 8 per group). On Days 0 and 13 (2 hours after the drug treatment), blood samples were collected for blood chemistry and PTH. Aortic rings were precontracted with phenylephrine (PE, 3 *μ*M), and the endothelium-dependent vasodilator acetylcholine was added in half-log increments (1 nM–30 *μ*M) at 3–5 minute intervals. Afterwards, aortic rings were precontracted with PE (3 *μ*M) and subsequently treated with endothelial-independent vasodilator sodium nitroprusside (SNP, 1 nM–1 *μ*M) at 3–5 minute intervals. (a) Serum PTH. (b) Serum Ca. (c) Acetylcholine-evoked relaxation. (d) SNP-evoked relaxation.

To investigate whether serum PTH suppression mediated by paricalcitol may have resulted in improved endothelial function in the uremic rats, we took an indirect approach and tested cinacalcet, a calcimimetic known to suppress PTH via interacting with the calcium sensing receptor independent of VDR. Consistent with the other two studies (Figures 2 and 3), the serum creatinine and BUN levels were elevated in the 5/6 NX rats compared to SHAM rats, which were not significantly affected by cinacalcet treatment at 30 mg/kg for 13 days (data not shown). Cinacalcet at 30 mg/kg after 13 days of treatment resulted in a decrease in serum PTH (a 66.8% reduction versus before treatment, Figure 4(a)) and total serum Ca (a 23% reduction, Figure 4(b)). Cinacalcet significantly elevated serum phosphorus by 18% (versus before treatment; data not shown). Figure 4(c) shows that the maximal aorta relaxation response to acetylcholine for the cinacalcet-treated group was −41.8 ± 4.2% with an EC_50 _ of 202 nM, which was not significantly different from the uremic 5/6 NX vehicle-treated group. As shown in Figure 4(d), while there was no significant difference in SNP-induced endothelial-independent relaxation between SHAM and uremic rats, cinacalcet exhibited a modest effect on reducing the vascular relaxation produced by SNP. These results suggest that PTH suppression is not linked to endothelial function improvement in the uremic rats and the effect of paricalcitol on improving endothelial function may be independent of PTH suppression.

### 3.4. L-NAME Abolished the Effect of Paricalcitol on Improving Endothelial Function.

**Figure 5:**
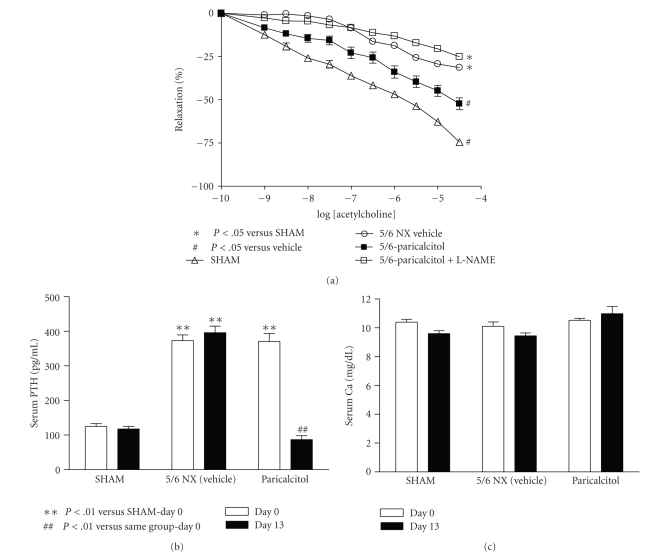
L-NAME abolished the effect of paricalcitol on improving endothelial dysfunction in 5/6 nephrectomized rats. SHAM and 5/6 NX rats were treated with vehicle or paricalcitol at 0.16 *μ*g/kg as described in “Materials and Methods” (*n* = 9–11 per group). Aortic rings were preincubated with 100 *μ*M L-NAME for 1 hour prior to the addition of PE to induce contraction, and the endothelium-dependent vasodilator acetylcholine was added in half-log increments (1 nM–30 *μ*M) at 3–5 minute intervals. (a) Acetylcholine-evoked relaxation. (b) Serum PTH. (c) Serum Ca.

To further investigate how VDR activation improves endothelial function, we tested the effect of L-NAME. In this particular study, the maximal aorta relaxation response to acetylcholine in SHAM rats was −74.5 ± 3.6% with an EC_50_ of 157 nM. Relaxation was significantly reduced in uremic 5/6 NX vehicle-treated rats (−31.4 ± 4.7% with an EC_50 _ of 358 nM). As shown in Figure 5(a), paricalcitol (0.16 *μ*g/kg, i.p., 3x/weeks for two weeks) increased the maximal relaxation response to acetylcholine to −52.3 ± 9.6% (*P* <.05) with an EC_50_ at 368 nM. Addition of L-NAME at 100 *μ*M during the aorta ring assay completely abolished the effect of paricalcitol on improving endothelial function. In this study, the serum PTH level was significantly reduced while the serum calcium level was not altered by paricalcitol (Figures 5(b) and 5(c)).

## 4. Discussion

Previously it has been shown that, in spontaneously hypertensive rats (SHR) with impaired endothelial function, oral cholecalciferol (vitamin D_3_) treatment significantly improved the endothelium-dependent vascular relaxation and hyperpolarization induced by acetylcholine [26]. Wong et al. [27] have also shown that calcitriol acutely reduced endothelium-dependent contractions in the aorta of the spontaneously hypertensive rat. Karavalakis et al. [28] reported that, in the 5/6 nephrectomized rats fed a special diet that induced severe hyperphosphatemia, paricalcitol at 0.2 *μ*g/kg reduced vasoconstriction but increased calcification in the large artery. As a comparison, our results show that paricalcitol improves endothelial function in the 5/6 NX rats with normal serum phosphate levels. 

More importantly, our data demonstrate that paricalcitol does not affect blood pressure in the uremic rats during the treatment period, suggesting that the effect of paricalcitol on improving endothelial function is independent of blood pressure control. Our observation is consistent with the finding made by Bodyak et al. [29] that paricalcitol attenuated the development of left ventricular abnormalities in the Dahl salt-sensitive rat (a different experimental model from the 5/6 nephrectomized uremic rats) without a significant effect on blood pressure. Karavalakis et al. [28] have also reported that, in the 5/6 nephrectomized rats fed a special diet that induced severe hyperphosphatemia, paricalcitol at 0.2 *μ*g/kg did not affect blood pressure. In a study reported by Mizobuchi et al. [23], 5/6 nephrectomized uremic rats developed significant hypertension, which was not affected by paricalcitol (0.8 *μ*g/kg) during a 4-month treatment period, while enalapril effectively lowered blood pressure. However, it is worth mentioning that, in the study by Freundlich et al. [30], the systemic blood pressure, which increased in all groups with renal ablation (versus SHAM rats), was significantly lower in rats treated with paricalcitol at either 0.1 or 0.3 *μ*g/kg. At this point it is not known why the results were different from different laboratories on a seemly similar uremic rat model. Hypertension is closely linked to endothelial dysfunction [8]. Many drugs that improve endothelial function also control hypertension, making it difficult to delineate the effects of these drugs on these two parameters. Like CKD patients, the 5/6 NX rats have severe hypertension and endothelial dysfunction. The observation from this study implies that it is possible to improve endothelial function in CKD independent of blood pressure control. 

In this study, due to the short duration of the treatment (2 weeks), paricalcitol didnot show an effect on serum creatinine and BUN. A study by Freundlich et al. [30] reported that the plasma creatinine level, elevated in all groups with renal ablation, started to decline after two weeks of paricalcitol (0.1 and 0.3 *μ*g/kg) treatment in the 5/6 nephrectomized uremic rats, eventually reaching levels comparable to SHAM rats and significantly lower than in nontreated 5/6 NX rats. Previously it has been shown [23] that, during a 4-month treatment period following nephrectomy, the increase in serum creatinine was less in the paricalcitol (0.8 *μ*g/kg) group than in the untreated group, although the difference failed to reach a statistical significance. 

Elevated PTH has been shown to play a role in various cardiovascular disorders including abnormal vasodilation [31]. Previously it has also been demonstrated that, in patients with primary hyperparathyroidism, parathyroidectomy significantly improved endothelial vasodilatory function [32]. The current study shows that PTH suppression is not linked to endothelial function improvement in the uremia rat since cinacalcet, a calcimimetic, does not show a significant effect on improving endothelial function although it suppresses PTH effectively. These data are of interest as they are consistent with the clinical observations that the survival benefit of VDR activation therapy in hemodialysis patients is observed across different serum PTH levels [14, 15]. 

We noticed the inconsistent effects of paricalcitol on aortic relaxation from study to study as evident in Figures 1 and 5. Paricalcitol at 0.083 *μ*g/kg in Figure 1 study almost normalized endothelial function to the SHAM level, while paricalcitol at 0.16 *μ*g/kg in Figure 5 study only partially improved endothelial function. A few possibilities exist for this discrepancy. Paricalcitol at a higher dose may decrease local activity of calcitriol or paricalcitol via CYP24 induction. Paricalcitol at the higher dose can also increase vascular calcification, which leads to reduced aortic relaxation. Alternatively, it may be the result of the inconsistent effects of paricalcitol on serum calcium in different batches of 5/6 nephrectomized uremic rats. It is of interest to note that changes in the serum calcium levels may affect endothelial function. Calcium is required for the activity of nitric oxide synthase, and thus, increasing extracellular or intracellular calcium concentrations may stimulate nitric oxide (NO) production. An increase in serum calcium may also alter the intracellular calcium level, leading to opening of endothelial calcium-sensitive potassium channels (K(+) channels) and vessel relaxation. Jolma et al. [33] reported that high-calcium diet enhanced resistance artery relaxation in the 5/6 NX rats likely mediated by K(+) channels. This effect was independent of the degree of renal impairment and blood pressure, but was associated with improved calcium metabolism because plasma levels of PTH and phosphate were decreased and serum calcium was increased. At the dose range of 0.021–0.16 *μ*g/kg that was used in this study, paricalcitol sometimes raises serum calcium; the effects vary from studies to studies and the causes for this variation is not well understood. For example, Noonan et al. [34] have shown that paricalcitol at 0.083 and 0.167 *μ*g/kg raised serum calcium levels while Wu-Wong et al. [35] reported that paricalcitol at 0.17 *μ*g/kg did not affect serum calcium levels. As shown in Figures 1 and 2, paricalcitol at 0.083 *μ*g/kg raised serum calcium and completely normalized endothelial function to the SHAM level. As a comparison, paricalcitol at 0.167 *μ*g/kg (Figure 5) did not raise serum calcium and only partially normalized endothelial function. Consistent with this notion, since serum calcium was significantly lower in the cinacalcet study (Figure 4), we cannot rule out the possibility that the lack of effect of cinacalcet on endothelial function may be due to the decrease in serum calcium levels after cinacalcet treatment. 

Borges et al. [26] have shown that, in the SHR rat model, cholecalciferol improved endothelial function likely mediated by endothelium-derived hyperpolarizing factor, but not nitric oxide. However, mounting evidence has shown that the systemic NO deficiency is the principal cause for endothelial dysfunction in CKD [36]. In a clinical study on hemodialysis patients, Passauer et al. [37] reported that endothelial dysfunction in uremic patients as determined by endothelium-dependent vasodilation in response to increased doses of acetylcholine is mainly attributed to reduced NO activity. Yamamizu et al. [38] have also reported that the decrease of NO production was the key factor linked to endothelial dysfunction in the 5/6 NX rats. Our data support the role of NO in CKD that L-NAME completely abolished the effect of paricalcitol on improving endothelial function in the uremic rats. The results suggest that paricalcitol treatment restores the systemic NO activity in the 5/6 uremic rats. Beside decreased nitric oxide generation, the accumulation of asymmetric dimethylarginine (ADMA, an endogenous nitric oxide inhibitor) is also known to contribute to endothelial dysfunction. We did a search but did not find any published work on VDR activation and ADMA. Although it is beyond the scope of this study, preliminary data from employing DNA microarray technology to analyze the aortic gene expression profiling in the 5/6 NX rats show that uremia is associated with abnormal expression of genes involved in increasing oxidative stress and paricalcitol treatment normalizes many of these genes [39]. Very little is known about VDR and oxidative stress. We hope the observations made in this study will stimulate others to investigate further about the role of VDR in regulating NOS, ADMA, and other factors involved in oxidative stress. 

In summary, VDR activation by paricalcitol significantly improves endothelial function in the 5/6 NX uremic rats. More importantly, the effect of paricalcitol is independent of blood pressure control and the serum PTH levels. Although randomized clinical studies are needed to confirm the cardiovascular and survival benefit of VDR activation therapy in CKD patients, data from the current study provide an important new insight that VDR activation improves endothelial function in uremia, which may be one of the mechanisms responsible for the cardiovascular benefit associated with these drugs in CKD.
